# New Insight into Cavitation-Assisted Chemical Refining and Enzymatic Modification of Vegetable Oils and Their Impact on Physicochemical Properties of Final Products

**DOI:** 10.3390/foods15030439

**Published:** 2026-01-25

**Authors:** Katsiaryna Kalenchak, Lucie Nováková, Tereza Váchalová, Tereza Honzíková, Tomáš Hybner, Aleš Rajchl, Helena Čížková, Iveta Šístková, Vojtěch Kružík, Markéta Berčíková, Jan Kyselka

**Affiliations:** 1 Department of Dairy, Fat and Cosmetics, Faculty of Food and Biochemical Technology, University of Chemistry and Technology, Technická 3, 166 28 Prague, Czech Republic; alisheva@vscht.cz (K.K.); lucie.novakova@vscht.cz (L.N.); tereza.vachalova@vscht.cz (T.V.); tereza.honzikova@vscht.cz (T.H.); tomas.hybner@vscht.cz (T.H.); 2 Department of Food Preservation, Faculty of Food and Biochemical Technology, University of Chemistry and Technology, Technická 3, 166 28 Prague, Czech Republic; ales.rajchl@vscht.cz (A.R.); helena.cizkova@vscht.cz (H.Č.); iveta.sistkova@vscht.cz (I.Š.); vojtech.kruzik@vscht.cz (V.K.)

**Keywords:** cavitation technologies, alkali neutralization, enzymatic interesterification, ultrasound, chemical refining, vegetable oil

## Abstract

The present study evaluates the impact of cavitation on the performance of the chemical refining of rapeseed oils and the enzymatic interesterification of fat blends using a powerful UP400S ultrasonicator (400 W, 20 kHz). Ultrasound-assisted alkali neutralization achieved efficiency comparable to that of the conventional 60 min process in only 7 min, with similar refining losses (5.04–6.80 wt.%), although slightly higher lipid peroxidation was observed. Performing the ultrasound cavitation under a protective nitrogen atmosphere minimized the formation of lipid peroxides and their breakdown products (i.e., hexanal, nonanal), partially protected tocopherols, and improved oxidative stability (IP at 120 °C = 3.9–4.4 h). Ultrasound-assisted enzymatic interesterification (EIE) of palm kernel fat and a palm stearin blend catalyzed by immobilized lipases (Lipozyme TL IM, Lipozyme RM IM, Novozyme 435) was carried out for the first time. Cavitation accelerated triacylglycerol rearrangement, reduced reaction time from 6 h (9.0·10^−3^ to 1.6·10^−2^ min^−1^) to only 1 h (5.5·10^−2^ to 1.2·10^−1^ min^−1^), and significantly affected melting point stabilization and solid fat content profile. In summary, ultrasound cavitation substantially enhanced mass transfer and reaction kinetics, demonstrating strong potential for process intensification in the edible oil industry. Further optimization of reaction conditions is required before large-scale industrial implementation.

## 1. Introduction

Cavitation technologies offer significant advantages over conventional refining methods in edible oil processing. In particular, cavitation-assisted refining—especially during deep degumming and bleaching stages—can enhance oil yield, reduce processing time, and lower the overall consumption of chemicals, washing water, steam, and energy [1].

Several studies have demonstrated the potential of ultrasound cavitation to improve the performance of the deep degumming process compared to conventional methods using dynamic mixers. Nosenko et al. (2024) reported that ultrasound-assisted water degumming of sunflower oil increased oil yield by 2.0–2.5% and achieved comparable removal of glycerophospholipids within 5–11 min, whereas conventional water degumming required up to 60 min [2]. Wu et al. (2024) showed that in the case of soybean oil, ultrasound-assisted water degumming reduced phosphorus content to 31.2 mg/kg compared to the 57.1 mg/kg achieved by conventional water degumming [3]. Mahmood-Fashandi et al. (2017) further confirmed that ultrasound-assisted acid degumming of both soybean and sunflower oils reduced phosphorus levels to 6.5–10.0 mg/kg depending on the processing time and temperature, markedly lower than acid degumming alone (13.9 mg/kg) [4].

The significance of ultrasound becomes even more apparent when considering that the improvement in glycerophospholipid removal is primarily due to the separation of non-hydratable species, which are more resistant to conventional refining methods. For example, the increase in deep degumming efficiency from 79.1% to 83.1% observed by More and Gogate (2018) [5] is particularly notable, as nearly the entire gain is attributed to the non-hydratable fraction. When the same authors combined ultrasound treatment with Fenton reagent, glycerophospholipid removal reached 98.3%. In addition, improvements in oxidative stability were observed, with excellent an TOTOX value of 1.2.

Several studies have evaluated ultrasound-assisted bleaching as an alternative to conventional processes across various edible oils. In rice bran oil, Zhang et al. (2024) [6] reported that ultrasound bleaching reduced carotenoids to only 5.9 mg/kg, compared to 8.6 mg/kg after conventional treatment. On the other hand, ultrasound bleaching led to stronger decreases in tocopherols (316 mg/kg vs. 333 mg/kg for conventional) and a reduction in linoleic acid content, suggesting cavitation-induced autoxidation of lipids.

In the case of rapeseed oil, De Jesús-Hernández et al. (2023) found that ultrasound bleaching removed 98% of chlorophylls and reduced carotene content by approximately 30%, comparable to conventional adsorptive bleaching, but with a 40% lower temperature, 67% shorter processing time, and 33% lower consumption of bleaching earth [7]. Similarly, Abedi et al. (2015) [8] demonstrated in soybean oil that ultrasound-assisted bleaching reduced carotenoids by approximately 82% and chlorophylls by approximately 95%, while also decreasing the content of phytosterols and tocopherols (e.g., campesterol 471.9 mg/kg vs. 490.7 mg/kg for control; γ-tocopherol 386.4 mg/kg vs. 498.5 mg/kg). The peroxide value was slightly higher for ultrasound-bleached oil compared to the control sample (1.4 meq. act. O/kg vs. 0.8 meq. act. O/kg), yet the method still reduced bleaching clay usage, temperature, and processing time by 35%, 35%, and 10%, respectively.

Icyer and Durak (2018) further demonstrated that ultrasound bleaching in canola oil can decrease processing temperature by 25% and duration by 50%, without significant changes in free fatty acid content [9]. However, both conventional and ultrasound-assisted bleaching increased lipid oxidation over time, as reflected by the formation of conjugated dienes and trienes, highlighting the importance of limiting exposure time. In olive oil, Jahouach-Rabai et al. (2008) [10] found that ultrasound-assisted bleaching did not alter the overall fatty acid composition. However, the presence of volatile oxidation products (e.g., hexanal, nonanal, etc.) and significant losses in total phytosterols (514 → 440 mg/kg) and α-tocopherol (140 → 56 mg/kg) were observed with longer duration and higher temperatures.

Ultrasound cavitation has also shown promising potential to improve enzymatic interesterification of triacylglycerols for technical purposes. In biodiesel production, Subhedar and Gogate (2016) reported a reduction in enzyme usage from 6% to 3%, a decrease in reaction time from 24 h to 3 h, and an increase in yield from 90% to 96% when ultrasound was applied [11]. Zheng et al. (2012, 2013) demonstrated that ultrasound-assisted esterification of phytosterols and flavonoids halved reaction times without impairing enzyme activity [12,13]. Similarly, Wang et al. (2019) found that ultrasound not only accelerated the enzymatic interesterification of triacylglycerols, but also reduced the optimal reaction temperature by 10 °C [14].

Although ultrasound-assisted cavitation has been widely studied in various stages of oil processing—such as degumming and bleaching—its application in the chemical removal of free fatty acids during neutralization remains unexplored. To address this gap, the present study investigates the use of ultrasound-assisted cavitation in the course of alkali neutralization of rapeseed oil, comparing it with conventional chemical neutralization. In addition to evaluating the efficiency of free fatty acid removal, we examined the effect of cavitation on the subsequent refining steps (bleaching and deodorization) to assess changes in oxidative stability. Furthermore, each experiment was conducted both with and without a nitrogen atmosphere to evaluate the role of triplet oxygen on the overall oxidative stability.

A second objective of the study focused on ultrasound-assisted enzymatic interesterification, which has so far found only limited application in the food industry despite its potential for producing structured fats for products such as margarines. In this part of the work, three commercial immobilized lipases were tested for the interesterification of a binary fat blend, both with and without ultrasound treatment. The efficiency of the process was evaluated through solid fat content, triacylglycerol profiling, determination of peroxide value, and calculation of the kinetic pseudoconstant, allowing for a quantitative comparison between conventional and ultrasound-assisted methods.

## 2. Materials and Methods

### 2.1. Materials and Chemicals

Commercial palm kernel fat (PKF) and palm stearin with an iodine value of 34 g I_2_/100 g (PST IV34) were supplied by AAK Sweden AB (Karlshamn, Sweden). Rapeseed oil of standard quality was purchased from Viterra Czech s.r.o. (Ústí and Labem, Czech Republic). Oleic acid (technical-grade) with a free fatty acid composition of 4.59% C16:0, 1.88% C18:0, 75.12% C18:1, 15.30% C18:2, and 3.11% other fatty acids, was purchased from Lach-Ner company. The commercial enzymes used for the interesterification supplied by Novozymes Spain S.A. (Madrid, Spain) were as follows: Lipozyme TL IM (*Thermomyces lanuginosus*, Novozymes, silica gel immobilization, *sn*-1,3-specific activity), Lipozyme RM IM (*Rhizomucor miehei*, Novozymes, microporous anion-exchange resin immobilization, *sn*-1,3-specific activity) and Lipozyme 435 (*Candida Antarctica* B, Novozymes, macroporus acrylic resin immobilization, nonspecific activity). Standards of α-, γ-, and δ-tocopherylquinones were synthesized in our laboratory as previously described [15]. Analytical standards of di- and triacylglycerols were also synthesized in our laboratory [16]. All other reagents and solvents were of analytical grade.

### 2.2. Chemical Refining of Rapeseed Oil Using Ultrasound Cavitation and Conventional Conditions Performed with/Without Nitrogen Atmosphere

A sample of commercial rapeseed oil spiked with either low (RO1 with acid value of 0.910 mg KOH/g) or high (RO2 with acid value of 2.877 mg KOH/g) levels of free fatty acids was subjected to a chemical refining pathway including the following: (1a) alkali neutralization using ultrasound cavitation (USC) or (1b) conventional alkali neutralization procedure (CON), followed by (2) adsorptive bleaching and (3) deodorization of bleached rapeseed oils using short-path distillation.

The ultrasound-assisted cavitation experiments (1a) were performed in a Hielscher noise protection box using an Ultrasonicator UP400S (Hielscher Ultrasonics GmbH, Teltow, Germany) with a power of 400 W and the amplitude adjusted to 100% and an ultrasound frequency of 20 kHz. The sample of RO1/RO2 (400 g) was heated up to 70 °C and placed into a SONOPULS RZ 5 rosette cell (L × W × D: 90 × 90 × 243 mm) with 3 side arms enabling intensified circulation of rapeseed oil and alkali (Figure S1a). After starting sonication, a 5% NaOH solution in a 5% stoichiometric excess was added to the RO1/RO2 sample by micropipette for 2 min. The neutralization of free fatty acids with simultaneous ultrasonication took another 5 min. At the end of the USC process, the temperature of the reaction mixture reached 85 °C. After the neutralization was completed, the resulting soap was centrifuged (30 °C, 5000 rcf, 10 min). Deacidified oils were thoroughly washed with hot (70 °C) distilled water until a neutral pH was achieved and dried over anhydrous Na_2_SO_4_ before adsorptive bleaching.

The conventional alkali neutralization (1b) of rapeseed oil (400 g) was carried out in a 1 L glass beaker in a water bath set to 80 °C. A 5% NaOH solution (80 °C) in a 5% stoichiometric excess was added to RO1/RO2 at 80 °C. Effective distribution of lye solution was performed by a Silverson’s L4RT (Silverson Machines, Inc., East Longmeadow, MA, USA) mixer operated at 1500 min^−1^. After 7 min of high-shear mixing, distilled water (3%; *w*/*w*) was added using a Heidolph PZR 2021 (Schwabach, Germany) (70 min^−1^) overhead stirrer and the agglomerated soapstock was allowed to settle down. Conventional neutralization was completed after 60 min. Further processing of the rapeseed oil was identical. Additionally, both the conventional and ultrasound neutralization experiments were performed in the same manner using an inert nitrogen atmosphere (Figure S1b).

Next, neutralized RO1/RO2 was heated to 100 °C. After the addition of Tonsil Optimum 210FF (1 wt. %) bleaching earth, the vacuum was set to 100 hPa and the batch was stirred at 500 min^−1^ for 30 min. The bleaching process (2) was completed by filtering the spent bleaching earth. Afterwards, molecular distillation simulating the deodorization stage (3) was performed on a FilmDist SP200 HT (Pilodist GmbH, Meckenheim, Germany) laboratory film evaporator at a temperature of 230 °C and a pressure of 1.33 hPa. The acid value [17], peroxide value [18], and the content of conjugated dienes and trienes [19] and tocopherols [20] were determined in samples of crude, alkali neutralized, bleached, and deodorized rapeseed oils according to official methods. Determinations were performed in triplicate.

### 2.3. Determination of Oxidative Stability of Rapeseed Oils and Secondary Products of Unsaturated Fatty Acid and Tocopherol Autoxidation

The oxidative stability of rapeseed oil before, during, and after chemical refining was determined using a Rancimat 743 Metrohm apparatus (Herisau, Switzerland). Each oil sample (2.5 g ± 0.1 g) was transferred into a reaction tube and oxidized at a temperature of 120 °C under a 20 L/h air flow. Volatile oxidative products were collected in a measuring vessel with 60 mL of redistilled water and the conductivity of the water was continuously measured for an increase in conductivity during the analysis. A second derivation of the Rancimat oxidation curve provided induction time. Determinations were performed in triplicate.

The HS-SPME analysis of the rapeseed oil samples (2.0 g) was performed in sealed 15 mL screw-top vials with PTFE/silicone septa (Supelco, Bellefonte, PA, USA) equipped with magnetic stirrers. DVB/CAR/PDMS 50 µm/30 µm fibre was exposed to headspace for 2 min at 40 °C. The analysis was performed on an Agilent gas chromatograph 7820A GC and a 5975 Series MSD (Agilent Technologies, Santa Clara, CA, USA). The target compounds were desorbed into the GC injector for 2 min at 240 °C. The volatile products were separated on a 30 m HP-5MS capillary column (Agilent Technologies) of dimensions 0.32 mm × 30 m, with a film thickness of 0.25 µm. The conditions of the analysis were as follows: splitless injection; flow of carrier gas (He) 0.9 mL/min; the temperature in the oven was programmed as follows: 40 °C (7 min); 40–200 °C (5 °C/min). Detection by the mass detector was performed in scan mode. The source and quadrupole temperatures were 230 and 150 °C, respectively, and the *m*/*z* ratio was 20 to 500. Concentrations of hexanal and nonanal were calculated from the calibration curves. Determinations were performed in triplicate.

The abundance of tocopherylquinones was determined by GC/FID method. First, 5.0 g of rapeseed seed oil was spiked with 1 mL of internal standard solution of 5α-cholestane (1 mg/mL) and saponified under reflux for 1 h in the dark under an argon atmosphere using 70 mL of ethanolic solution of 1 M KOH. After the addition of 100 mL of distilled water, the unsaponifiable compounds were extracted three times with 100 mL of diethyl ether. Combined organic phases were washed in a separatory funnel with 40 mL of distilled water and 40 mL of 0.5 M KOH solution and several times with distilled water to reach a neutral pH value. Analyses of target compounds were performed using an Agilent 6890N gas chromatograph (Agilent Technologies, USA) coupled with a flame-ionization detector and a 30 m HP-5MS capillary column (Agilent Technologies) of 0.32 mm × 30 m, with a film thickness of 0.25 μm. The conditions of analysis were as follows: ethyl acetate solution (≈1%) was used for the injection (1 μL), with a split injection (1:25) at 300 °C; flow of carrier gas (He) 1.0 mL/min; the temperature in the oven was programmed as follows: 80 °C (1 min), 80–320 °C (15 °C/min), and 320 °C (20 min); flame-ionization detection at 320 °C, flow of H_2_ 40 mL/min, air flow 450 mL/min, and make-up gas (N_2_) flow 45 mL/min. Determinations were performed in triplicate. Correction factors for tocopherylquinones were used before conversion of peak areas into mass percentages. GC/MS (EI) was recorded using an Agilent 7820A GC system and a 5975 Series mass selective detector (Agilent Technologies, Santa Clara, CA, USA) under the same conditions used for GC/FID.

### 2.4. Enzymatic Interesterification of Fat Blends Using Ultrasound Cavitation and Conventional Conditions

Commercial samples of palm kernel fat and palm stearin IV 34 were used as substrates for either the (1a) ultrasound-assisted or (1b) conventional enzymatic interesterification. Prior to the acyl exchange, both fats were dried under vacuum (10 hPa) at 120 °C for 2 h. The composition of acylglycerols and fatty acids was summarized in Tables S1 and S2, respectively.

Ultrasound-assisted cavitation (USC) experiments (1a) were performed in a Hielscher noise protection box using an Ultrasonicator UP400S (Hielscher Ultrasonics GmbH, Germany) with a power of 400 W, the amplitude adjusted to 100%, and an ultrasound frequency of 20 kHz. In each trial, the fat blend (200 g, 1:1, *w*/*w*) was melted at 70 °C and placed into a SONOPULS KG3 (BANDELIN electronic GmbH & Co. KG, Berlin, Germany) cooling vessel connected to a water bath set to 53 °C (Figure S2, upper image) above the slip melting point of the reaction mixture (melting point of the mixture was 51.05 °C). After starting sonication, 8 wt. % of enzyme was immediately added and the interesterification reaction was carried out for 60 min. Three types of enzymes were used: Lipozyme TL IM, Lipozyme RM IM, and Novozyme 435. At given intervals of 10, 20, 30, 40, 50, and 60 min, filtered aliquots (5 mL) were taken for the analyses of solid fat content (SFC), melting point, distribution of acylglycerols, acid value, and peroxide value.

Conventional (CON) enzymatic interesterification (1b) was performed in a three-neck flask using the same three enzymes as in experiments 1a. In each experiment, 200 g of the dried fat blend (1:1, *w*/*w*) was heated to the required reaction temperature according to optimal reaction temperature of the enzymes (50 °C for Lipozyme RM IM, 65 °C for Novozyme 435, and 70 °C for Lipozyme TL IM) under a stream of argon (Figure S2, lower image). Once the desired temperature was reached, 8 wt. % of the appropriate lipase was immediately added. The reaction was allowed to proceed for 6 h. At given intervals of 10, 20, 30, 40, 50, 60, 120, 180, 240, and 300 min, filtered aliquots (5 mL) were taken for the analyses of SFC, melting point, distribution of acylglycerols, acid value, and peroxide value. Melting points and SFC profiles were determined according to AOCS Cc 3-25 and ISO 8292-1 official methods [21,22]. Each measurement was performed in duplicate.

Enzymatic interesterification was thought to be a pseudo-first-order reaction [23] for which the following differential velocity equation applies:

(1) $$-\frac{{dc}_{TAG}}{dt}=k\cdot c_{TAG}$$

After integration, relation (2) was obtained for the calculation of kinetic parameters:

(2) $$\ln\frac{c_{{TAG}_{0}}}{c_{TAG}}=k\cdot t$$

where *c*TAG is the TAG concentration at time *t* [mol/L], *c*TAG_0_ is the initial TAG concentration [mol/L], *t* is the reaction time [min], and *k* is the pseudo-first-order rate constant [min^−1^].

### 2.5. Determination of Triacylglycerol Composition and Fatty Acid Profile Before and After Enzymatic Interesterifications

Analysis of triacylglycerols was performed on an Agilent 8890N gas chromatograph (Agilent Technologies, USA) coupled with a flame-ionization detector and an Optima 17(TG) column (Supelco, Bellefonte) of dimensions 0.32 mm × 25 m and a film thickness of 0.1 µm. The conditions of the analysis were as follows: a hexane solution of TAG (1%) was used for the injection (1 µL), with split injection (1:25) at 300 °C; flow of carrier gas (He) 1.5 mL/min; the temperature in the oven was programmed as follows: 80 °C (1 min), 80–250 °C (20 °C/min), 250–370 °C (5 °C/min), and 370 °C (20 min); flame-ionization detection (FID) at 380 °C, flow of H_2_ 40 mL/min, air flow 450 mL/min, and make-up gas (N_2_) flow 45 mL/min. Determinations were performed in duplicate.

The analysis of fatty acid methyl esters was performed on an Agilent 8890N gas chromatograph (Agilent Technologies, USA) coupled with a flame-ionization detector and an SP 2560 capillary column (Supelco, Bellefonte) of dimensions 0.25 mm × 100 m and a film thickness of 0.2 µm. The conditions of the analysis were as follows: a hexane solution of FAME (1%) was used for the injection (1 µL), with split injection (1:50) at 220 °C; carrier gas flow (He) 1 mL/min; analysis at 175 °C for 120 min; FID at 250 °C, flow of H_2_ 40 mL/min, air flow 450 mL/min, and make-up gas (N_2_) flow 45 mL/min. Determinations were performed in duplicate.

### 2.6. Statistical Analysis

The results of all experiments are presented as the mean ± standard deviation. Statistical analysis of the acquired data was conducted using Statistica 14 software (TIBCO Software Inc., San Ramon, CA, USA). The statistically significant effect of ultrasound and conventional technologies was evaluated using one-way analysis of variance. All statistical analyses were conducted at a significance level of α = 0.05; therefore, differences with *p* ≤ 0.05 were considered statistically significant.

## 3. Results and Discussion

### 3.1. The Impact of Ultrasound Cavitation on the Performance of Chemical Refining of Rapeseed Oils and Their Quality

The application of cavitation reactors represents a current commercial innovation in the processing of vegetable oils by chemical refining, particularly in the stage of alkali neutralization of free fatty acids [24]. In addition, hydrodynamic cavitation is also industrially used during the deep degumming stage to remove hydratable and non-hydratable glycerophospholipids [25]. Alkali neutralization and deep degumming occur at the interfacial boundary, and thus, their efficiency as well as their refining losses strongly depend on the size of the dispersed heavy-phase particles. Conventional rotor–stator dynamic mixers cannot further increase interfacial area, which is why hydrodynamic cavitation reactors have been successfully applied in the modern edible oil industry. From an economic perspective, the residence time of the oil in the apparatus and, above all, the overall refining losses are of particular importance for manufacturers. On the other hand, cavitation technologies can negatively affect the quality of refined edible oils, leading to the conclusion that further systematic investigation is warranted [24,25].

Since operating hydrodynamic cavitation in a laboratory setting is challenging, ultrasound cavitation (USC) experiments were conducted instead. These were performed in the unique batch rosette vessel using a UP400S ultrasonicator (Hielscher Ultrasonics GmbH, Teltow, Germany) with a power of 400 W, an amplitude set to 100%, and an ultrasound frequency of 20 kHz, to simulate cavitation-assisted alkali neutralization. The aim was to compare ultrasonic cavitation (7 min) with the conventional (CON) alkali neutralization of rapeseed oil (60 min), which is generally limited by the diffusion of NaOH through the layer of resulting soaps according to Fick’s law. Our model system consisted of fully refined rapeseed oil enriched with technical-grade oleic acid (75.12% C18:1, 15.30% C18:2, 4.59% C16:0, 1.88% C18:0, and 3.11% others), resulting in two input oils: RO1 and RO2, with FFA contents of 0.457 wt.% (AV = 0.910 mg KOH/g) for RO1 and 1.446 wt.% (AV = 2.877 mg KOH/g) for RO2. Due to cavitation phenomena and the exothermic nature of the neutralization reaction, the temperature of the reaction mixture during USC increased from an initial 70 °C (upon insertion of RO1/RO2 into the rosette vessel) to a final temperature of 85 °C, with a residence time of only 7 min.

**Figure 1:**
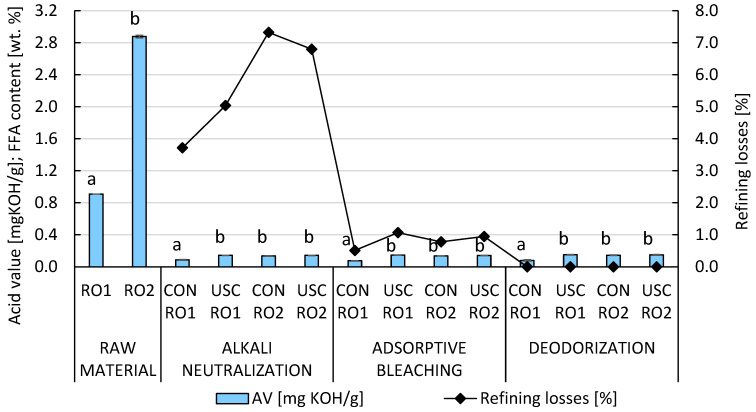
The impact of conventional and ultrasound-assisted chemical refining of RO1 and RO2 without nitrogen atmosphere on the removal of FFA and the overall refining losses. Data represent means ± standard deviation. Different superscript letters in the columns of particular technology stages indicate significant differences (*p* ≤ 0.05) among samples. RO1, rapeseed oil with 0.457 wt. % of free fatty acids; RO2, rapeseed oil with 1.446 wt. % of free fatty acids; USC, ultrasound cavitation-assisted chemical refining; CON, conventional chemical refining.

The efficiency of RO2 deacidification, which had a high FFA content, was comparable between the conventional method (95.3%) and cavitation technology (95.0%) (Figure 1). In the case of the higher-quality RO1, deacidification was more effective using the conventional setup, achieving 90.4% FFA removal. Acoustic cavitation technology led to similar losses of acylglycerols (5.04–6.80 wt.%) compared to the conventional batch experiment (3.72–7.32 wt.%). Thin-layer chromatography (TLC) screening of the soapstock composition revealed significant losses of acylglycerols, also referred to as “neutral oils”, in micelles after both processing methods as a result of occlusion and inclusion phenomena. Refining losses observed in the cavitation process can be attributed to the use of a 5% excess of NaOH relative to FFA, which likely promoted undesirable saponification of ester bonds at elevated temperatures (70–85 °C). In accordance with good manufacturing practice, NaOH solution should be dosed stoichiometrically when using (hydrodynamic) cavitation reactors. However, to ensure comparability, identical conditions were maintained for both experimental approaches.

**Figure 2:**
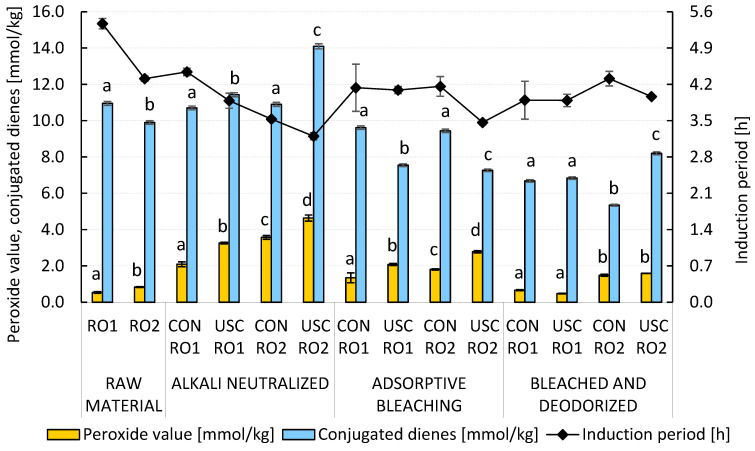
The impact of conventional and ultrasound-assisted chemical refining of RO1 and RO2 without nitrogen atmosphere on the formation and decomposition of hydroperoxides, conjugated dienes, and induction period. Data represent means ± standard deviation. Different superscript letters above the PV and CD content in particular technology stages indicate significant differences (*p* ≤ 0.05) among samples. USC, ultrasound cavitation-assisted chemical refining; CON, conventional chemical refining.

A key aspect of the refining process is the oxidative stability of fully refined rapeseed oil. To assess this, we monitored the content of hydroperoxides and conjugated dienes (CD) along with the determination of the induction period (IP) of the RO1 and RO2 samples using a Rancimat 743 device at 120 °C. As shown in Figure 2, ultrasound-assisted alkali neutralization led to slightly increased formation of lipid hydroperoxides (3.26–4.64 mmol/kg) and conjugated dienes (11.43–14.10 mmol/kg). In the presence of good hydrogen donors such as α- and γ-tocopherols, the group of CD represented either the hydroperoxides with a conjugated double bond system (19.5–32.9 mol. %) formed upon kinetic stage of autoxidation, or the group of alkenals, unsaturated carbonyl compounds with a conjugated double bond (67.1–81.5 mol. %). Further bleaching with acid activated bleaching earth- and deodorization-induced acid- and homolytically catalyzed decomposition of hydroperoxides into volatile secondary autoxidation products. As a result, both the peroxide value (0.48–1.59 mmol/kg) and the concentration of CD (5.34–8.20 mmol/kg) decreased in the fully refined rapeseed oils. It is worth noting that the content of hydroperoxides in the fully refined samples RO1 and RO2 was comparable to that of RBD soybean oil, which was also prepared using ultrasound cavitation technologies in the study by Abedi et al. (2015) [8].

### 3.2. The Impact of Protective Atmosphere on Tocopherols and Oxidative Stability of Rapeseed Oils Obtained by Ultrasound-Assisted and Conventional Chemical Refining

**Figure 3:**
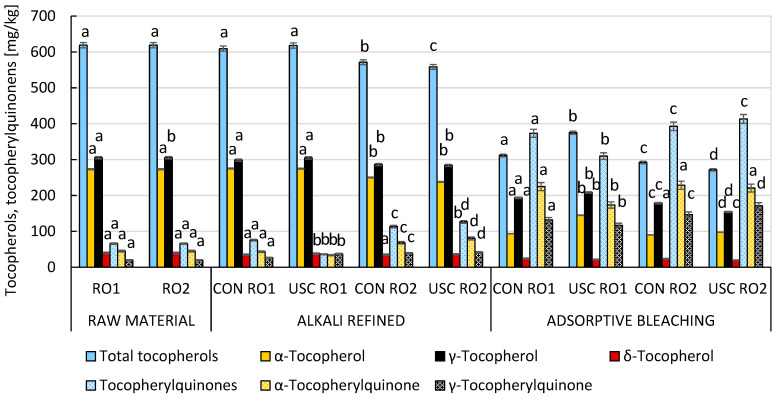
The impact of conventional and ultrasound-assisted chemical refining of RO1 and RO2 without nitrogen atmosphere on the technological losses of tocopherols and the formation of tocopherylquinones. Data represent means ± standard deviation. Different superscript letters above the content of the studied compounds in particular technology stages indicate significant differences (*p* ≤ 0.05) among samples. USC, ultrasound cavitation-assisted chemical refining; CON, conventional chemical refining.

Chemical refining generally improved the induction periods of both samples (3.9–4.3 h using a Rancimat 743 device at 120 °C) compared to their quality after the alkali neutralization stage (Figure 2). In the RO2 sample with higher acidity, tocopherol losses (7.7–10.8%) after alkali neutralization could be attributed to their adsorption at the interfacial boundary and subsequent entrapment in micelles along with soaps (Figure 3). Further technological losses during chemical refining resulted mainly from two-electron oxidation of tocopherols by reactive oxygen species, leading to a marked increase in the concentration of tocopherylquinones (TQ) in the fully refined rapeseed oil. Losses of tocochromanols during gentle deodorization by molecular distillation were practically negligible under the conditions applied (230 °C, 1.33 hPa, no stripping medium). The highest TQ content was observed in the USC-treated RO2 sample (Figure 3). As expected, the fully refined RO2 sample contained the highest levels of hydroperoxides and “core aldehydes” (residual CD) (Figure 2). Logically, the same sample contained the lowest levels of tocopherols (271.6 mg/kg), yet the induction period was not significantly reduced (4.0 h). The conversion of α-tocopherol and γ-tocopherol with different degrees of substitution of the tocochromanol system to the corresponding α-tocopherylquinone (47.0–68.5%) and γ-tocopherylquinone (23.7–46.0%) was critical. As a result, we decided to conduct identical technological experiments in the protective nitrogen atmosphere and compare them with previous experiments in which a nitrogen atmosphere was not used (Section 3.1).

**Figure 4:**
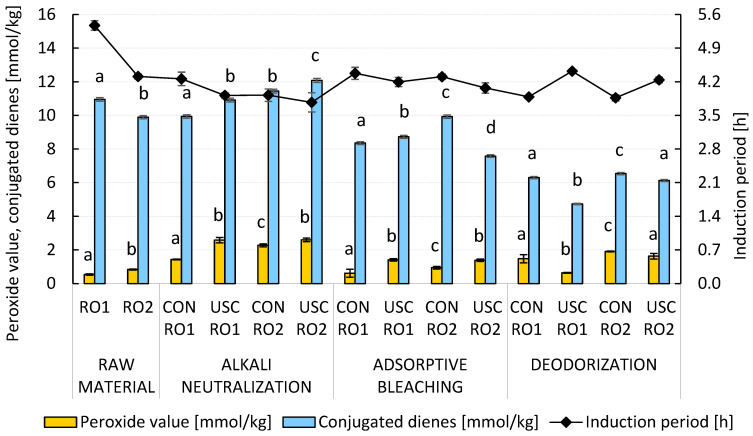
The impact of conventional and ultrasound-assisted chemical refining of RO1 and RO2 in a protective nitrogen atmosphere on the formation and decomposition of hydroperoxides, conjugated dienes, and induction period. Data represent means ± standard deviation. Different superscript letters above the PV and CD content in particular technology stages indicate significant differences (*p* ≤ 0.05) among samples. USC, ultrasound cavitation-assisted chemical refining; CON, conventional chemical refining.

As shown in Figure S3, the introduction of nitrogen into the neutralization reaction significantly increased the refining losses of the RO1 (6.2–7.2 wt.%) and RO2 (11.0–13.2 wt.%) samples. In both cases, losses were higher in the cavitation-assisted technology as a result of more intense emulsification of rapeseed oils and the corresponding saponification of acylglycerols at the newly formed interfacial boundary. However, the efficiency of the deacidification stage did not change significantly. The peroxide value after alkali neutralization with nitrogen did not exceed 2.60 mmol/kg, which is a significant improvement compared to previous experiments without protective atmosphere (Figure 2). Similarly, we observed a slight increase in the overall oxidation stability of the fully refined samples of RO1 and RO2 with the induction period of the edible oils at 120 °C of 3.9 to 4.4 h (Figure 4).

**Figure 5:**
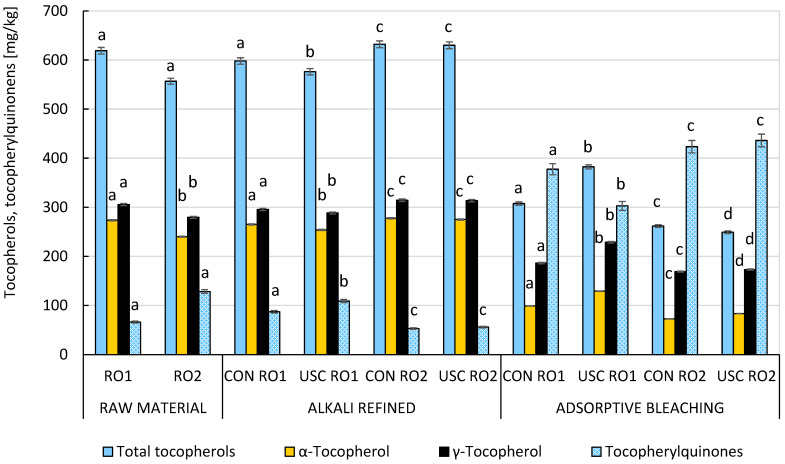
The impact of conventional and ultrasound-assisted chemical refining of RO1 and RO2 under inert conditions on the losses of tocopherols and the formation of tocopherylquinones. Data represent means ± standard deviation. Different superscript letters above the content of the studied compounds in particular technology stages indicate significant differences (*p* ≤ 0.05) among samples. USC, ultrasound cavitation-assisted chemical refining; CON, conventional chemical refining.

The violent collapse of cavitation bubbles caused their implosion, according to the reversed Bernoulli’s principle, resulting in a drastic increase in both pressure (up to 1000 atm.) and temperature (up to 5000 °C). On the other hand, the pulsation and implosion of bubbles thoroughly mixed the oil and heavy water phase and thus improved the liquid–liquid interfacial area [24,25]. Despite refining losses, collapse of cavitation bubbles in an atmosphere deprived of oxygen prevented losses of key lipophilic antioxidants (tocopherols). However, this protective effect was apparent only after alkali neutralization (Figure 5). In contrast, the resulting contents of tocopherols (249–382 mg/kg) and tocopherylquinones (303–435 mg/kg) in the fully refined RBD rapeseed oils were quite comparable, regardless of whether a protective atmosphere was used.

Adsorptive bleaching of rapeseed oils previously deacidified under a nitrogen atmosphere differed significantly, with low levels of hexanal and nonanal, which belong to markers of the autoxidation of the dominant linoleic and oleic acids. Both compounds belong to breakdown products of hydroperoxides derived from *n*-6 and *n*-9 fatty acids and are sensorially active with relatively low perception thresholds. In the case of neutralization processes performed without nitrogen, the contents of hexanal and nonanal, as shown in Table 1, increased by 31.8–68.1 μmol/kg and 22.0–42.3 μmol/kg, respectively. In contrast, adsorptive bleaching reduced the content of hydroperoxide precursors by 810–1330 μmol/kg, whereas the contents of hexanal and nonanal, as shown in Table 1, increased by only 16.6–48.0 μmol/kg and 13.2–28.2 μmol/kg, respectively. This clearly demonstrates that the transformation of hydroperoxides yields other key volatile compounds (i.e., propanal, pentane, hexane, (*E*)-2-butenal, (*E*)-2-pentenol, (*E*)-2-pentenal, pentanal, heptane, pentanol, (*E*)-2-hexenal, heptanal, (*E*)-2-heptenal, (*E*,*E*)-2,4-heptadienal, octanal) as identified by the SPME-GC/MS analysis, as well as carbonyl products bound in acylglycerols, the so-called core aldehydes. Although volatile compounds were quantitatively removed by molecular distillation, the core aldehydes remain in the oils and pose a potential dietary risk for consumers.

### 3.3. The Impact of Ultrasound Cavitation on the Performance of Enzymatic Interesterification of Fat Blends and Their Quality

Another important innovation in edible oil processing is the shift from conventional alkali-catalyzed interesterification, mainly using sodium methoxide, to more sustainable interesterification catalyzed by lipases. Both enzymatic interesterification (EIE) and conventional alkali neutralization are limited by insufficient reaction rates due to diffusion constraints. In the case of alkali neutralization, the rate-limiting step is the diffusion of NaOH through the layer of fatty acid soaps. In heterogeneous enzymatic catalysis, the limitation arises from the internal diffusion of the substrate to the active site of the biocatalyst, which is typically immobilized on a commercial carrier. In both cases, diffusion represents the bottleneck of the process, and cavitation-assisted technologies offer promising solutions to overcome these limitations.

For our purposes, commercially immobilized nonspecific (Novozyme 435) and 1,3-regiospecific (Lipozyme RM IM, Lipozyme TL IM) lipases were employed as biocatalysts to carry out interesterification reactions using palm stearin IV34 (1.0 equiv.) and palm kernel fat (1.0 equiv.). The standard quality parameters of the resulting blend were represented by an acid value of 0.65 mg KOH/g, a peroxide value of 2.56 meq. act. O/kg, and a melting point of 51.05 °C. The EIE was performed under non-aqueous, ultrasound-assisted conditions at 53 °C for only 1 h using a UP400S ultrasonicator. The aim was to compare USC-assisted enzymatic interesterification with a conventional EIE setup conducted at 50–70 °C for 6 h, depending on the optimal temperature of the selected lipase. It was assumed that these reaction times were sufficient to reach thermodynamic equilibrium.

**Figure 6:**
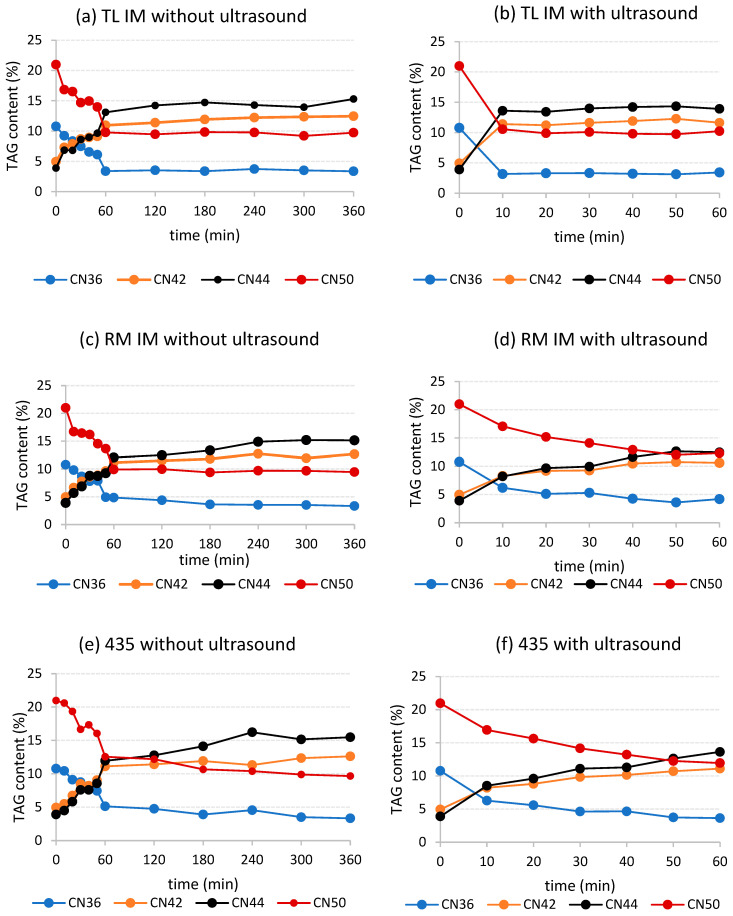
Content of selected TAGs during enzymatic interesterification using (**a**) Lipase TL IM, conventional arrangement; (**b**) Lipase TL IM, ultrasound treatment; (**c**) Lipase RM IM, conventional arrangement; (**d**) Lipase RM IM, ultrasound treatment; (**e**) Lipase 435, conventional arrangement; (**f**) Lipase 435, ultrasound treatment. CN—carbon number.

In commercial PST IV34 obtained by dry fractionation of palm oil, the most prominent TAGs were those with a carbon number (CN) of 50 (41.88%), as shown in Table S1. 1,3-Dipalmitoyl-2-oleoylglycerol (POP), 1,2-dipalmitoyl-3-oleoylglycerol (PPO), and 1,3-dipalmitoyl-2-stearoylglycerol (PSP), together with minor trisaturated (SSS-type) triacylglycerols, constitute the major components of the TAG CN50 group, reflecting the high content of palmitic (59.56%), oleic (26.91%), and stearic (5.51%) acyls (Table S2) [16]. In the case of PKF, the TAG distribution was significantly shifted toward lower-molecular-weight species, with TAG CN36 (20.99%) represented mostly by 1,2,3-trilauroylglycerol (LLL) as the major component (Table S1). Both TAG groups with CN36 (10.77%, 168.5 mmol/kg) and CN50 (20.97%, 251.0 mmol/kg) also prevailed in the final fat blend used as the model substrate for the EIE reactions. During EIE, these species underwent intermolecular rearrangement with the most abundant fatty acyls (C12:0 22.05%, C14:0 8.83%, C16:0 35.18%, C18:1 21.89%) on the glycerol backbone. With respect to the TAG and fatty-acyl profiles, we decided to monitor the progress of the EIE reaction only by the decrease in TAGs with CN36 and CN50, along with the increase in TAGs with CN42 (i.e., LLO, LLS, MPL) and CN44 (i.e., PPL, LOM, LSM, MPM), resulting from the enzymatic exchange of laurate, myristate, palmitate, and oleate fatty-acyl moieties (Figure 6).

When evaluating the reaction catalyzed by the Lipozyme TL IM, it could be seen that the most significant changes in the content of key TAG species occurred within 60 min under conventional conditions (Figure 6a). In contrast, Lipozyme TL IM interesterification assisted by ultrasound cavitation was completed within the first 10–20 min (Figure 6b). This fact is a novel finding with a great potential for industrial improvements in the field of EIE. A similar trend was observed for EIE reactions enzymatically catalyzed by regiospecific Lipozyme RM IM and nonspecific Novozyme 435, where the greatest fatty-acyl exchange occurred during the first 50 min of the USC-assisted EIE reaction, and within 180–240 min under conventional conditions (Figure 6c–f). To sum up, when using a combination of Lipozyme TL IM and a powerful USC sonotrode, thermodynamic equilibrium was clearly achieved within 20 min, whereas with Lipozyme RM IM and Novozyme 435, equilibrium was nearly achieved. The degree of triacylglycerol conversion also differed. Under conventional conditions, 68.7–69.1% of TAG CN36 and 53.6–54.5% of TAG CN50 were transformed by EIE, while under ultrasound treatment the values were 61.1–68.2% for TAG CN36 and 41.3–51.2% for TAG CN50, respectively. This raises the question of whether the ultrasound-assisted reaction affects enzyme selectivity and the potential for consecutive acyl migration to the *sn*-2 position. The next step was to evaluate the kinetics of both EIE processes by calculating the pseudo-first-order rate constant, where a higher pseudo-rate constant indicated a faster reaction. As shown in Table 2, the application of ultrasound significantly accelerated the enzymatic interesterification of TAG CN36 (5.5·10^−2^ to 1.2·10^−1^ min^−1^)—by approximately one order of magnitude—compared to conventional conditions (9.0·10^−3^ to 1.6·10^−2^ min^−1^). Our conclusions were in good agreement with the results of Subhedar and Gogate (2016) and Zheng et al. (2012, 2013), who reported an up to 8 times acceleration of EIE for technical (methanolysis) and synthetic purposes (phytosterol and flavonoid esters) [11,12,13].

The advantage of conventional EIE is the reuse of the lipase with minimal impact on its activity. In the case of ultrasound-assisted EIE, we observed slight destruction of the commercial carriers as well as the reduction in the catalytic activity of enzymes similar to Wang et al. (2019) [14], especially in the case of lipases immobilized on both porous resins. Catalyst turnover is a serious problem and it will therefore be necessary to find a technological reaction arrangement that will ensure the stability of commercial lipases and their reuse in the near future, while also offering manufacturers significant time savings. This drawback has to be solved prior commercial application of cavitation techniques connected with EIE. In this respect, application of lipases immobilized on silica gel combined with hydrodynamic cavitation appears to be a less-destructive process and thus, more suitable for real application.

### 3.4. The Impact of Ultrasound Cavitation on Physical Properties of the Interesterified Fat Blends

**Figure 7:**
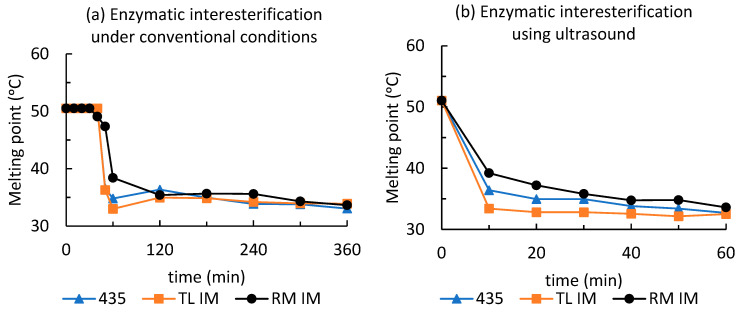
Melting points of fat blends during enzymatic interesterification: (**a**) under conventional EIE arrangement, (**b**) using ultrasound-assisted EIE. Data represent means ± standard deviation.

EIE can significantly affect the physical properties of TAGs by modulating their profiles and thereby altering the melting behaviour of interesterified fat blends. The melting point is an important parameter for evaluating the reaction progress of both conventional and ultrasound-assisted EIE. As the amount of high-melting TAGs decreased during EIE, the melting point of the reaction mixture also decreased. Triacylglycerols with high CN values from 48 to 52 accounted for 48.81% (Table S1) of the original fat blend, which had a melting point of 51.1 °C. Under conventional conditions, the melting point dropped sharply within the first 40–60 min of the EIE reaction for all enzymes (Figure 7a), despite their different specificity. Not surprisingly, the steep decrease in melting point from 51.1 °C to 36.3 °C observed for Lipozyme TL IM correlated with the formation of lower-melting TAGs with CN42 and CN44 and, conversely, with the disappearance of TAGs with CN36 and CN50 (Figure 6). In contrast, acyl exchange catalyzed by the second sn-1,3-specific lipase, Lipozyme RM IM, stabilized the melting point at 35.4 °C after 120 min. When using the nonspecific lipase Novozyme 435, a melting point of 36.6 °C was reached after 50 min of reaction (a decrease of 14.5 °C), showing a trend similar to that observed for TL IM. As shown in Figure 6a,c,e, changes in TAG composition occur from the very beginning of the reaction; however, changes in melting point during conventional interesterification did not become apparent until after 40–50 min (Figure 7a). Ultrasound-assisted enzymatic interesterification progressed more rapidly, with a significant decrease in melting point observed for all mixtures after just 10 min (Figure 7b). However, a constant melting point within 10–20 min was reached only for the mixture catalyzed by Lipozyme TL IM. For mixtures catalyzed by the other enzymes, melting points continued to stabilize throughout the full 60 min reaction period. The decrease in melting point, similar to overall EIE progress, was an order of magnitude faster than in the conventional process (Table 2).

**Figure 8:**
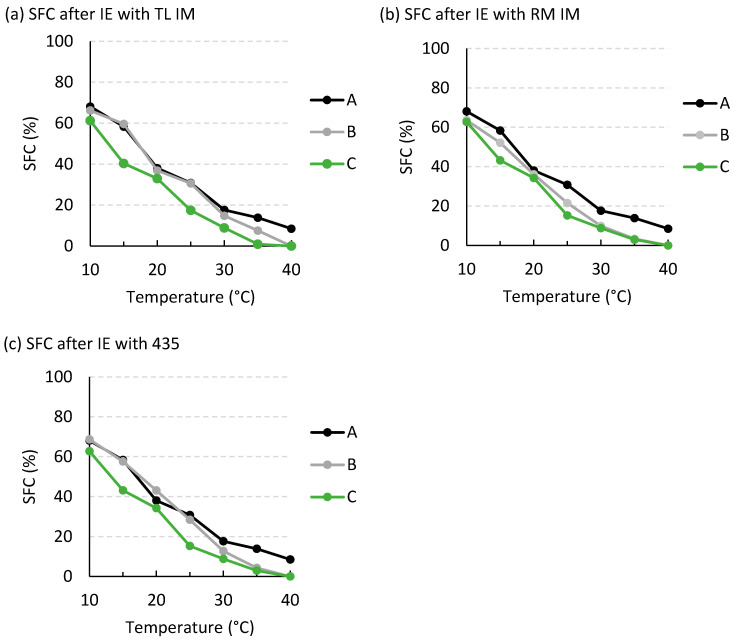
Solid fat content of fat blends. (**a**) Material before the interesterification reaction; (**b**) product after 6 h of the reaction under conventional arrangement; (**c**) product after 1 h of the reaction using ultrasound treatment. Data represent means ± standard deviation.

At the end of the EIE reactions, the solid fat contents (SFCs) of the resulting blends were also determined (Figure 8). All blends exhibited the desired values at the key temperature points: more than 10% solids at 20 °C and less than 10% at 35 °C, as previously described by Oliveira et al. (2017) and Xu et al. (2018) [26,27]. At 35 °C, the structured fat prepared using cavitation showed a lower solids fraction than that obtained under conventional conditions (1–3% versus 3–8%). A lower SFC value is preferred in this context, as demonstrated in the study by Dian et al. (2007) [28]. As shown, enzymatic interesterification modified the SFC profile, reflecting changes in the composition of individual triacylglycerols (Figure 6). The differences are particularly pronounced before and after the reaction under ultrasound treatment (Figure 8c). It should be emphasized that the SFC profile was measured for fats after only 1 h of reaction, compared with 6 h required for conventional interesterification. Therefore, it can be concluded that the application of ultrasound significantly shortens, and thus accelerates, the course of interesterification as well as crystallization phenomena.

An important finding was that, under the given conditions, the use of ultrasound increased the peroxide value in some of the EIE reactions. The mixture before the reaction had a peroxide value of 2.56 meq. act. O/kg. After the reaction catalyzed by Lipozyme TL IM, the value increased only slightly to 2.93 meq. act. O/kg, whereas reactions catalyzed by Lipozyme RM IM and Novozyme 435 showed substantially higher peroxide values of 11.27 and 20.04 meq. act. O/kg, respectively. These results indicate that the peroxide values of the latter two mixtures were excessively high and thus unsatisfactory according to Codex Alimentarius recommendations. Both lipases were immobilized on micro- and macro-porous carriers and it is highly likely that residual triplet oxygen trapped in their pores contributed to cavitation-assisted lipid peroxidation. An important conclusion is not only the need to select an enzyme based on its specificity and stability under cavitation-assisted EIE conditions, but also to consider the influence of the carrier material itself. We have demonstrated that enzymes immobilized on silica gel are the preferred choice when using cavitation technology.

## 4. Conclusions

**Figure 9:**
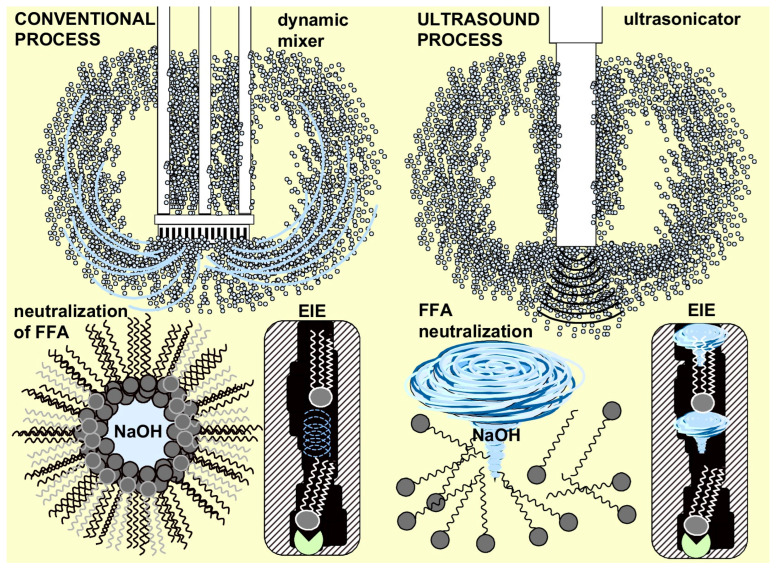
Schematic illustration of processing differences between alkali neutralization of free fatty acids and enzymatic interesterification of fat blends under the conditions of conventional mixing (**left**) compared with cavitation technologies (**right**).

Both chemical and enzymatic technologies were limited by insufficient reaction rates due to either the diffusion constraints of NaOH through the layer of soaps or by the internal diffusion of triacylglycerols to the active site of the lipase immobilized on the commercial carrier (Figure 9). Our conclusions confirmed that cavitation-assisted technologies offer a promising solution to overcome alkali neutralization limitations by breaking up agglomerates (micelles). In the case of EIE, microjets and turbulent flows disrupted stagnant boundary layers, transported triacylglycerols from fat blends to the lipase’s active site faster, and accelerated the removal of the final products—structured triacylglycerols. Moreover, USC-assisted neutralization provided soapstock with lower losses of acylglycerols as a result of their occlusion and inclusion phenomena.

The use of 7 min USC in the course of the chemical refining of rapeseed oils significantly accelerated technologically important reactions, including alkali neutralization, reaching a residual FFA content of 0.149–0.151 mg KOH/g as well as an undesirable autoxidation of unsaturated fatty acids (0.48–1.59 meq. act. O/kg) and the associated two-electron degradation of tocopherols. The application of nitrogen partially protected tocopherols and inhibited the formation of hexanal (16.6–48.0 μmol/kg) and nonanal (13.2–28.2 μmol/kg) with other volatile compounds, but at the same time increased overall chemical refining losses (6.2–13.2 wt. %). Nevertheless, cavitation technologies have shown considerable potential in the chemical refining of vegetable oils and fats. It is important to dose NaOH according to stoichiometry, optimize the residence time of reactants in the apparatus, and ensure that the acid value of the raw material does not exceed 1.5 mg KOH/g. Otherwise, application of a physical refining pathway is preferred.

Enzymatic modification of triacylglycerols resulted in approximately a sixfold acceleration of acyl exchange between TAG (5.5·10^−2^ to 1.2·10^−1^ min^−1^) compared to conventional EIE technology (9.0·10^−3^ to 1.6·10^−2^ min^−1^). The decrease in melting point curve showed similar acceleration to EIE kinetics. USC-assisted technology positively affected the SFC profile of PST IV34 and PKF blend with less solid fraction at 35 °C. We demonstrated that the immobilized lipase carrier is crucial, as porous materials with triplet oxygen residues inside pores enhanced the autoxidative changes in the final fat blend with the resulting peroxide value of 11.27 and 20.04 meq. act. O/kg and also led to easier enzyme inactivation. In the case of the Lipozyme TL IM with silica gel carrier, the resulting oxidative level was acceptable (2.93 meq. act. O/kg), while lipase stability and catalyst turnover significantly improved.

## Figures and Tables

**Table 1:** The impact of conventional and ultrasound-assisted alkali neutralization with and without a protective nitrogen atmosphere and further bleaching of RO1 and RO2 on the formation of hexanal and nonanal from *n*-6 and *n*-9 polyunsaturated fatty acids. Data represent means ± standard deviation. Different superscript letters in columns indicate significant differences (*p* ≤ 0.05) among samples.

Chemical Refining	Hexanal [μmol/kg]		Nonanal [μmol/kg]	
	Without N_2_	With N_2_ atm.	Without N_2_	With N_2_ atm.
USC RO1	54.7 ± 1.9 ^a^	48.0 ± 0.9 ^b^	34.9 ± 1.5 ^a^	28.2 ± 1.7 ^b^
CON RO1	31.8 ± 0.6 ^a^	16.6 ± 0.6 ^b^	22.0 ± 0.3 ^a^	13.2 ± 0.7 ^b^
USC RO2	68.1 ± 0.9 ^a^	41.2 ± 1.2 ^b^	42.3 ± 2.0 ^a^	28.1 ± 0.9 ^b^
CON RO2	51.8 ± 1.1 ^a^	24.3 ± 0.3 ^b^	35.9 ± 1.8 ^a^	26.6 ± 1.4 ^b^

**Table 2:** Pseudo-first-order rate constant *k* [min^−1^] determined for the TAG CN36 at the initial stage of the interesterification reaction.

Commercial Enzyme	Ultrasound EIE [min^−1^]	Conventional EIE [min^−1^]
Lipozyme TL IM	1.2 × 10^−1^	1.6 × 10^−2^
Lipozyme RM IM	5.5 × 10^−2^	9.0 × 10^−3^
Lipozyme 435	5.4 × 10^−2^	1.4 × 10^−3^

## Data Availability

The original contributions presented in this study are included in the article and Supplementary Materials; further inquiries can be directed to the corresponding author.

## Supplementary Materials

The following supporting information can be downloaded at https://www.mdpi.com/article/10.3390/foods15030439/s1, Table S1: Acylglycerol composition (TAG, DAG) of commercial palm kernel fat, palm stearin with iodine value of 34 g I_2_/100 g and their blend (1:1, *w*/*w*) determined by HT-GC/FID. Table S2: Fatty acid composition of commercial palm kernel fat, palm stearin with iodine value of 34 g I2/100 g and their blend (1:1, *w*/*w*) determined by GC/FID. Figure S1: Ultrasound-assisted alkali neutralization performed in SONOPULS RZ 5 rosette cell using an Ultrasonicator UP400S (Hielscher Ultrasonics GmbH, Germany) without (a) and in the protective atmosphere of nitrogen (b). Figure S2: Ultrasound-assisted EIE performed in duplicated reaction vessel cell using an Ultrasonicator UP400S (Hielscher Ultrasonics GmbH, Germany) (upper image) and conventional EIE in the protective atmosphere of nitrogen (lower image). Figure S3: The impact of conventional and ultrasound-assisted chemical refining of RO1 and RO2 in the protective atmosphere of nitrogen on the removal of FFA and the overall refining losses.

## Abbreviations

The following abbreviations are used in this manuscript:

PKF	Palm kernel fat
PST IV34	Palm stearin with an iodine value of 34 g I_2_/100 g
RO1	Rapeseed oil with 0.457 wt. % of free fatty acids
RO2	Rapeseed oil with 1.446 wt. % of free fatty acids
CON	Conventional chemical refining
USC	Ultrasound cavitation-assisted chemical refining
HS-SPME	Headspace solid phase microextraction
MSD	Mass spectrometry detector
EIE	Enzymatic interesterification
TL IM	*Thermomyces lanuginosus*, novozymes, silica gel immobilization, sn-1,3-specific activity
RM IM	*Rhizomucor miehei*, novozymes, microporous anion-exchange resin immobilization, sn-1,3-specific activity
SFC	Solid fat content
TAG	Triacylglycerol
CD	Conjugated dienes
TQ	Tocopherylquinones
