# Supplementary material for: New Insight into Cavitation-Assisted Chemical Refining and Enzymatic Modification of Vegetable Oils and Their Impact on Physicochemical Properties of Final Products
Source: Foods. 2026 Jan 25;15(3):439. doi: 10.3390/foods15030439 (PMC12897236; doi:10.3390/foods15030439)
Supplement: Supplementary file 1 [file foods-15-00439-s001.zip › foods-4058774-supplementary.pdf]

New Insight into Cavitation-Assisted Chemical Refining and Enzymatic Modification of Vegetable Oils and Their Impact on Physicochemical Properties of Final Products

Katsiaryna Kalenchak [1], Lucie Nováková [1], Tereza Váchalová [1], Tereza Honzíková [1], Tomáš Hybner [1], Aleš Rajchl [2], Helena Čížková [2], Iveta Šístková [2], Vojtěch Kružík [2], Markéta Berčíková [1,*] and Jan Kyselka [1,*]

[1] Department of Dairy, Fat and Cosmetics, Faculty of Food and Biochemical Technology, University of Chemistry and Technology, Technická 3, 166 28 Prague, Czech Republic; alisheva@vscht.cz (K.K.); lucie.novakova@vscht.cz (L.N.); tereza.vachalova@vscht.cz (T.V.); tereza.honzikova@vscht.cz (T.H.); tomas.hybner@vscht.cz (T.H.)

[2] Department of Food Preservation, Faculty of Food and Biochemical Technology, University of Chemistry and Technology, Technická 3, 166 28 Prague, Czech Republic; ales.rajchl@vscht.cz (A.R.); helena.cizkova@vscht.cz (H.Č.); iveta.sistkova@vscht.cz (I.Š.); vojtech.kruzik@vscht.cz (V.K.)

* Correspondence: marketa.bercikova@vscht.cz (M.B.); kyselkaj@vscht.cz (J.K.)

Table S1. Acylglycerol composition (TAG, DAG) of commercial palm kernel fat, palm stearin with iodine value of 34 g I_2/100 g and their blend (1:1, w/w) determined by HT-GC/FID.

**Acyglycerol profile**	**Palm kernel fat (wt. %)**	**Palm stearin IV34 (wt. %)**	**Fat blend to EIE (1:1, w/w, wt. %)**
ΣDAG	1.17	2.3	1.74
**TAG-CN30**	1.15	-	0.58
**TAG-CN32**	6.00	0.02	3.07
**TAG-CN34**	**8.25**	0.04	4.25
**TAG-CN36**	**20.99**	0.06	**10.77**
**TAG-CN38**	**16.00**	0.05	**8.32**
**TAG-CN40**	**9.87**	0.21	**5.30**
**TAG-CN42**	**9.79**	0.04	4,95
**TAG-CN44**	**7.60**	0.01	3.90
**TAG-CN46**	**5.25**	2.29	4.27
**TAG-CN48**	**5.31**	**28.43**	**16.72**
**TAG-CN50**	2.63	**41.88**	**20.97**
**TAG-CN52**	2.85	**20.06**	**10.97**
**TAG-CN54**	1.65	3.86	2,57
others	1.49	2.96	1.62

Table S2. Fatty acid composition of commercial palm kernel fat, palm stearin with iodine value of 34 g I_2/100 g and their blend (1:1, w/w) determined by GC/FID.

Fatty acid profile	Palm kernel fat (wt. %)	Palm stearin IV34 (wt. %)	Fat blend to EIE (1:1, w/w, wt. %)
**C6:0**	0.11	-	0.06
**C8:0**	2.53	-	1.27
**C10:0**	2.84	-	1.42
**C12:0**	**43.90**	0.20	**22.05**
**C14:0**	**16.48**	1.18	**8.83**
**C16:0**	**10.80**	**59.56**	**35.18**
**C18:0**	2.84	**5.51**	4.18
**C18:1 *trans***	0.67	0.63	0.65
**C18:1 *cis***	**16.87**	**26.91**	**21.89**
**C18:2 *trans***	-	0.19	0.10
**C18:2 *cis***	2.71	**5.35**	4.03
**C20:0**	0.14	0.38	0.26
**C18:3 *cis***	0.11	0.08	0.10

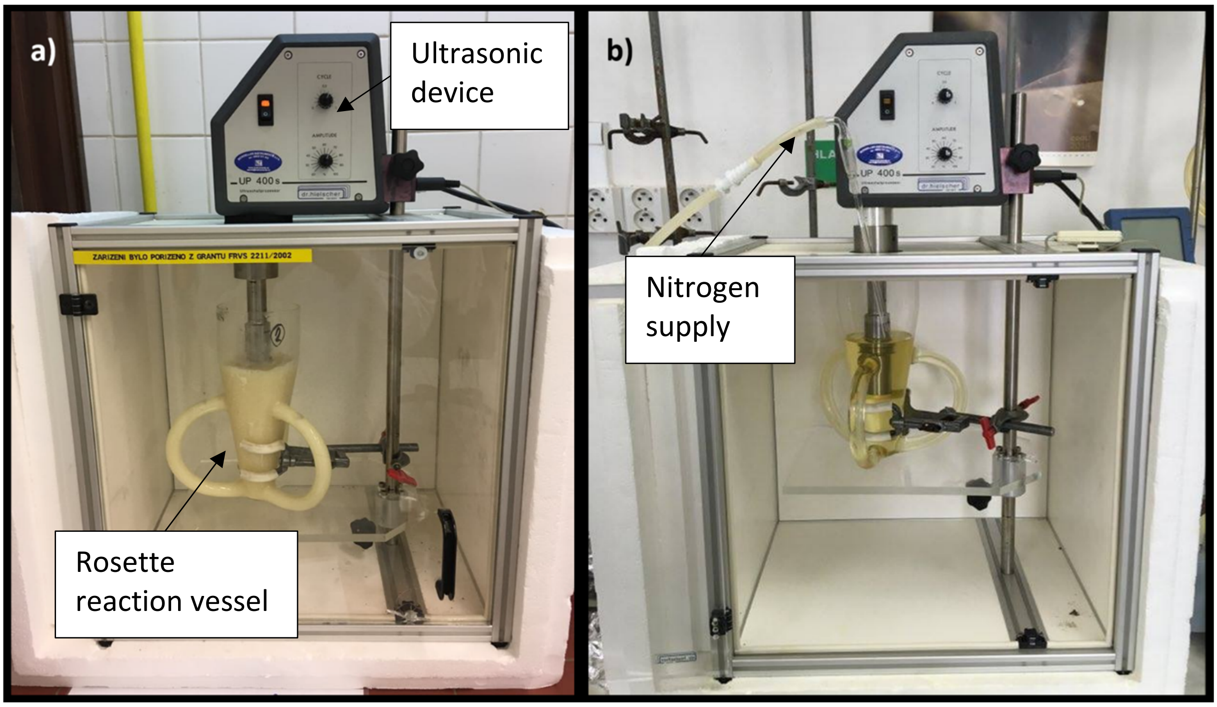

Figure S1. Ultrasound-assisted alkali neutralization performed in SONOPULS RZ 5 rosette cell using an Ultrasonicator UP400S (Hielscher Ultrasonics GmbH, Germany) without (**a**) and in the protective atmosphere of nitrogen (**b**).

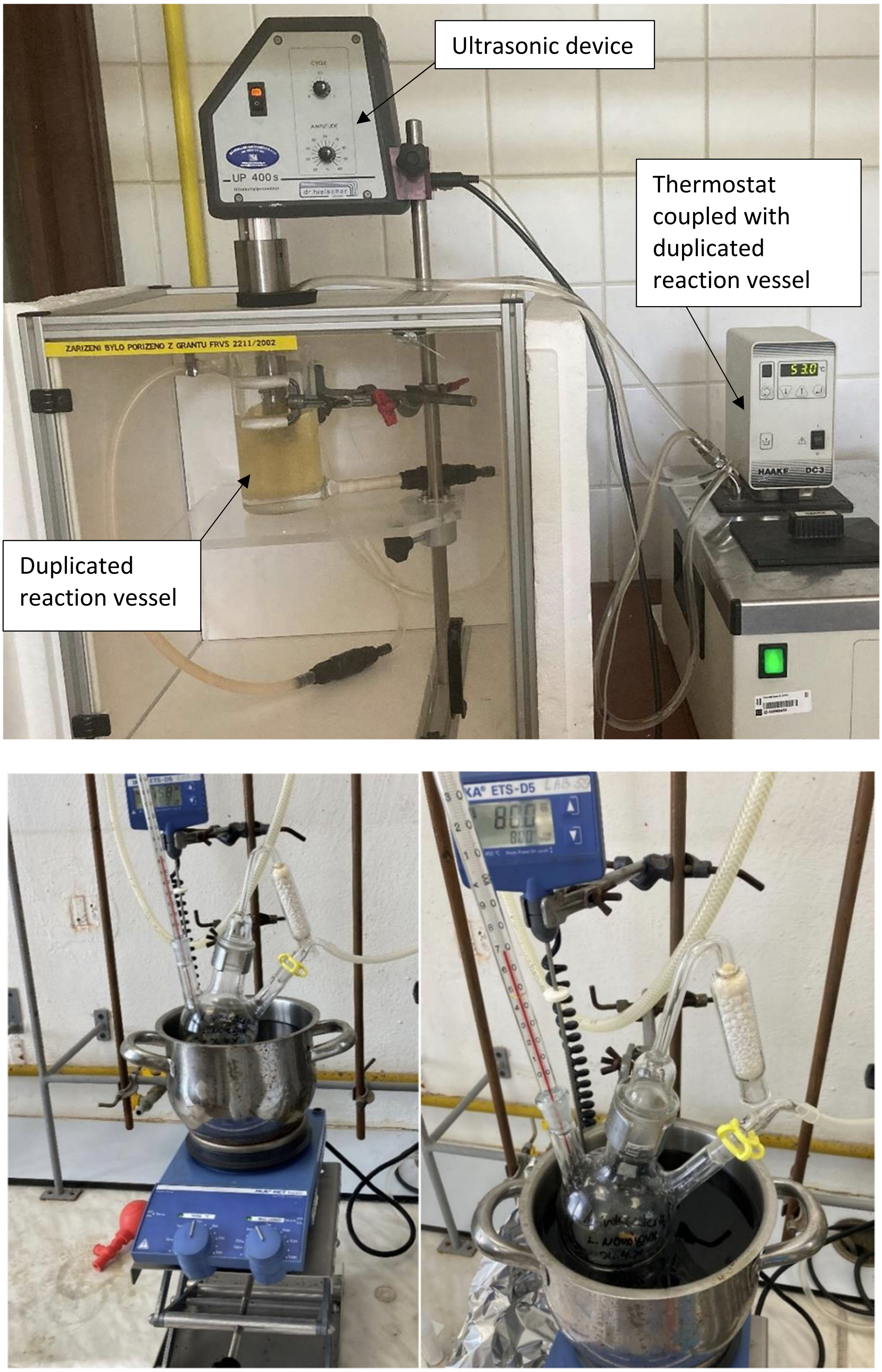

Figure S2. Ultrasound-assisted EIE performed in duplicated reaction vessel cell using an Ultrasonicator UP400S (Hielscher Ultrasonics GmbH, Germany) (**upper image**) and conventional EIE in the protective atmosphere of nitrogen (**lower image**).

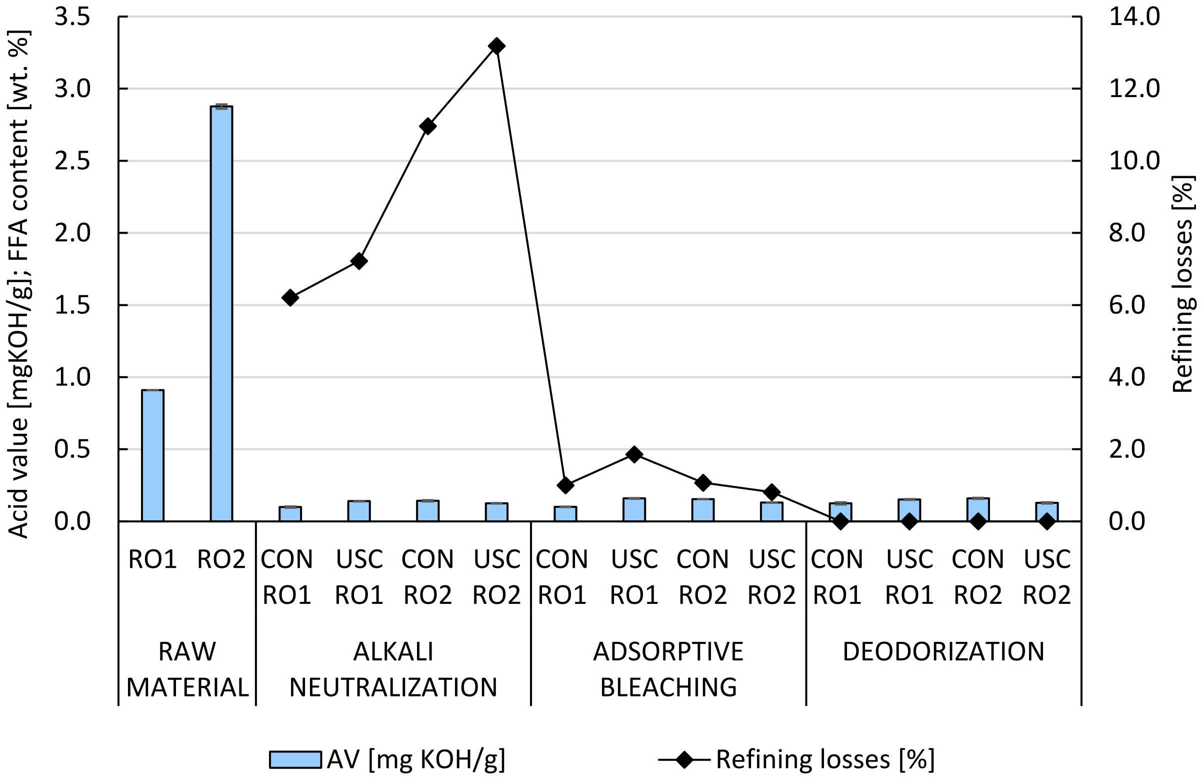

Figure S3. The impact of conventional and ultrasound-assisted chemical refining of RO1 and RO2 in the protective atmosphere of nitrogen on the removal of FFA and the overall refining losses. Data represent means ± standard deviation. Different superscript letters in the columns of particular technology stage indicate significant differences ($p \leq 0.05$) among samples. RO1, rapeseed oil with 0.457 wt. % of free fatty acids; RO2, rapeseed oil with 1.446 wt. % of free fatty acids; USC, ultrasound cavitation-assisted chemical refining; CON, conventional chemical refining.
